# Prevalence and correlates of HIV among men who have sex with men in Tijuana, Mexico

**DOI:** 10.7448/IAS.18.1.19304

**Published:** 2015-02-09

**Authors:** Eileen V Pitpitan, David Goodman-Meza, Jose Luis Burgos, Daniela Abramovitz, Claudia V Chavarin, Karla Torres, Steffanie A Strathdee, Thomas L Patterson

**Affiliations:** 1 Division of Global Public Health, Department of Medicine, University of California, San Diego, CA, USA; 2 Department of Psychiatry, University of California, San Diego, CA, USA; 3 Agencia Familiar Binacional, A.C., Tijuana, Mexico

**Keywords:** men who have sex with men, correlates of HIV infection, HIV prevalence, US–Mexico border, global public health, respondent-driven sampling

## Abstract

**Introduction:**

Men who have sex with men (MSM) in developing countries such as Mexico have received relatively little research attention. In Tijuana, Mexico, a border city experiencing a dynamic HIV epidemic, data on MSM are over a decade old. Our aims were to estimate the prevalence and examine correlates of HIV infection among MSM in this city.

**Methods:**

We conducted a cross-sectional study of 191 MSM recruited through respondent-driven sampling (RDS) in 2012. Biological males over the age of 18 who resided in Tijuana and reported sex with a male in the past year were included. Participants underwent interviewer-administered surveys and rapid tests for HIV and syphilis with confirmation.

**Results:**

A total of 33 MSM tested positive for HIV, yielding an RDS-adjusted estimated 20% prevalence. Of those who tested positive, 89% were previously unaware of their HIV status. An estimated 36% (95% CI: 26.4–46.5) had been tested for HIV in the past year, and 30% (95% CI: 19.0–40.0) were estimated to have ever used methamphetamine. Independent correlates of being infected with HIV were methamphetamine use (odds ratio [OR]=2.24, *p*=0.045, 95% CI: 1.02, 4.92) and active syphilis infection (OR=4.33, *p*=0.01, 95% CI: 1.42, 13.19).

**Conclusions:**

Our data indicate that MSM are a key sub-population in Tijuana at higher risk for HIV. Tijuana would also appear to have the highest proportion among upper-middle-income countries of HIV-positive MSM who are unknowingly infected. More HIV prevention research on MSM is urgently needed in Tijuana.

## Introduction

In Latin America, UNAIDS and other official bodies have reported a stable HIV/AIDS epidemic [1]. However, this masks epidemics that are more concentrated and dynamic in specific areas and among subpopulations such as men who have sex with men (MSM) [2–4]. While HIV prevalence in Mexico overall is low (<1%) [5], there are almost 180,000 people in the country living with HIV [6], making it the country with the second highest number of HIV cases in Latin America. Among MSM in Mexico, recent data from a national study using time–location sampling show a higher HIV prevalence (17%) than previously acknowledged [7].

In addition, higher HIV prevalence has been found in Mexican border cities, including Tijuana, which borders San Diego, California. The Tijuana–San Diego border crossing is the busiest in the world, recording more than 60 million northbound travellers in 2010 [8]. Tijuana has an HIV prevalence that is double the national average [2], and prevalence is highest among certain subgroups that include injection drug users (~4%) [9], female sex workers (~6%) [10] and their male clients (~5%) [11]. While these populations have been fairly well examined [10], surprisingly little is known about MSM. Identifying the prevalence and correlates of HIV among MSM in Tijuana is key to developing prevention strategies among this population and curbing the growing HIV epidemic in the US–Mexico border region.

To date, there are only two reports of HIV prevalence among MSM in Tijuana, both obtained from convenience samples and both dating back more than a decade. A study published in 1991 reported an HIV prevalence of 11.6% among MSM in Tijuana (*n*=233) [12], while a 2002 study of young MSM (18–29 years of age) in the same city reported an HIV prevalence of 18.9% (*n*=249) [13]. In order for governmental and non-governmental agencies to develop effective HIV prevention interventions for MSM in Tijuana, it seems imperative to obtain a more current estimate of prevalence and correlates of HIV infection in this population.

Correlates of HIV that have been observed elsewhere among MSM are likely to also be important in understanding HIV among MSM in Tijuana. Among these are methamphetamine (meth) use, HIV testing status and co-occurring sexually transmitted infections (STIs) [13–15]. Since Tijuana lies on a major drug trafficking route, illicit drugs are widely available and easily accessible. Meth is one of the most widely used drugs in Tijuana, along with heroin [16, 17]. HIV testing rates in the city are low [18], which leaves many individuals, including high-risk MSM, unaware of their HIV status. Finally, Tijuana has relatively high rates of STIs, which further increases vulnerability to HIV [19].

The goal of this study was to estimate HIV prevalence among MSM in Tijuana using sampling methods that can be generalized to the city's overall MSM population. Specifically, we used respondent-driven sampling (RDS) to recruit MSM and obtain population estimates. RDS is a sampling technique that uses peer recruitment, a dual incentive system and probability weights to account for non-random sampling biases. It has been proven effective for sampling from hidden populations [20]. We also aimed to identify correlates of HIV infection. Based on the literature, we hypothesized that meth use, HIV testing status and co-occurring STIs would be associated with HIV infection.

## Methods

### Study location and ethical approval

This cross-sectional study was conducted at the offices of the *Agencia Familiar Binacional* (AFABI), a community-based HIV and AIDS service organization (CBO) that is located in central Tijuana. Screening occurred at AFABI. Study staff explained the research purpose, study benefits and risks, and peer recruitment procedure to eligible participants. Those willing to participate provided written informed consent. All research methods were reviewed and approved by the institutional review boards of the University of California San Diego and the Universidad Autónoma de Baja California.

### Sampling

Participants were recruited through RDS between August 2012 and May 2013. Initially, six seeds were selected who were diverse with respect to age, area of residence within the city, ethnicity, socio-economic status and self-identified sexual orientation. These men had large networks of peers (>15 individuals) whom they were willing to recruit. At the mid-point of the study, four additional seeds, who were drawn from a pool of candidates identified by staff at AFABI, were included to boost recruitment.

Each seed was given three coupons to recruit other MSM. Each peer recruit was given the same number of coupons until a target sample size of 200 participants was reached and recruitment was stopped. All coupons had a three-week expiration date. Participants received $20 US for enrolling and $5 for each referral, for a maximum total of $35 US. All participants, ineligible candidates and visitors to the study site were offered free condoms and educational materials on HIV prevention.

### Eligibility criteria

Men were eligible to participate if they were (1) biologically male, (2) residents of Tijuana, (3) reported having oral or anal sex with another man in the past 12 months, (4) 18 years or older, (5) had a study coupon (except seeds) and (6) were willing to provide informed consent. Participants were excluded if (1) they had been previously enrolled in the study or (2) were obviously under the influence of alcohol or another drug at the time of enrolment.

### Data collection

Trained interviewers native to and trusted by the local community collected study-related data through face-to-face interviews using a standardized survey. We chose face-to-face interviews rather than computerized assessments because we wanted direct feedback about any issues with survey items (given the lack of previous research with this specific population). Interviewers queried participants on demographics, and sensitive topics, including substance use history, sexual behaviour, history of infection with STIs, knowledge of HIV and STI symptoms and transmission routes, experiences as MSM, encounters with the law and travel to the United States. Privacy was maintained by holding interviews in private rooms. Surveys took one to three hours depending on the participant's answers.

All participants were tested for HIV and syphilis infection using rapid tests. HIV testing was done on a finger-prick blood sample using the Advanced Quality HIV test by Intec Products, Inc. (Xiamen, China). All participants underwent pre- and post-test counselling according to Mexican national guidelines. A positive HIV result prompted a second rapid test. Those who tested positive on both rapid tests provided a venous blood sample that was sent to the San Diego County Public Health Laboratories (SDCPHL) for confirmation of HIV infection through immunofluorescence assay (IFA). Syphilis infection status was also confirmed by SDCPHL thru *Treponema pallidum* haemagglutination assay and rapid plasma reagin titers. A titer equal to or higher than 1:8 was considered positive for active syphilis. Confirmatory results for both HIV and syphilis were made available to participants within three weeks. All who were confirmed positive for HIV were referred for psychological support on-site and for medical treatment at Centros Ambulatorios de Prevencion y Atención en SIDA e ITS (CAPASITS), the Mexican national institute that provides no- or low-cost HIV care to infected individuals. Those testing positive for syphilis were treated on-site at no charge by AFABI's medical doctors according to national guidelines and were urged to follow up with RPR titers in six months [21].

### Measures

Demographics included age, gender, sexual orientation, living status and travel to or deportation from the United States. Drug use questions included types of substances ever used, routes of administration (ingested, injected, smoked or sniffed, as applicable), and frequency of use, if any, in the past month.

Substances queried were marijuana, heroin, inhalants (glue, gasoline), methamphetamines, ecstasy, cocaine, benzodiazepines, barbiturates, amyl nitrate (poppers), gamma-hydroxy butyric acid (GHB), ketamine or others, and possible combinations of these. Alcohol use was characterized through the Alcohol Use Disorder Identification Test (AUDIT), whereby a score of eight or more was considered to indicate hazardous alcohol use [22, 23].

Data on sexual behaviour in the past two months included types of activity (anal, oral and vaginal) with partners of the same or opposite sex, types of partner (regular, casual, anonymous, transactional), condom use with each type of partner and sexual practice, use of public venues for sexual encounters (night clubs, bars, cafes, steam baths, adult movie theatres, dark rooms – unlighted rooms, often attached to bars, where men have sex [24]; public places – parks, public restrooms; Internet cafes), and the use of drugs and alcohol before or during sex. Information was also collected on history of STI infection, knowledge about HIV and STIs, HIV testing history, condom access and use and circumcision status.

Personal network size was defined as the number of MSM whom the participant knew who were aged 18 and older and lived in Tijuana, with whom the participant had been in contact in the past month, and who the participant believed would have been likely to recruit him if provided with a coupon.

### Statistical analysis

Crude and RDS-adjusted prevalence estimates were calculated for the sample on demographic and HIV-risk-related variables. RDS-adjusted estimates were calculated using RDS Analysis Tool 7.1 (RDSAT) [25]. Parameters were set to “dual component” for average network estimation, 15,000 iterations for bootstrapping, alpha 0.025 and enhanced data smoothing. All subsequent analyses were performed on SPSS 21 (IBM, Chicago, IL).

We performed univariate analyses using unweighted data that compared HIV-positive to HIV-negative participants, using logistic regressions to identify variables as candidates for a multivariate model. After excluding seeds, as they were not randomly recruited, we calculated unadjusted odds ratios (OR) and 95% confidence intervals (95% CI) with univariate logistic regression and determined a multivariate model to identify independent correlates for HIV infection.

Because the creation of statistical models using RDS sample data is thought by some to lead to the underestimation of standard errors [26], we used a six-step process outlined by Spiller [27], which was recently used in another RDS study [28] to conduct multivariate analysis. This method proposes to adjust for homophily by entering recruiter-level characteristics into recruit-level regression models. It also recommends adjusting for respondent clustering by estimating fixed- or random-effects models if clustering is observed. First, we evaluated clustering by geographic area, recruitment chain and common-recruiter cluster levels using one-way ANOVA, but we found no significant clustering. Then, we determined which variables had high homophily (>0.15) in relation to our dependent variable (HIV infection status). We then built a respondent-level model. To determine independent predictors of HIV infection, we tested a multivariate model with all variables that attained significance of *p*<0.10 in the univariate analyses. Next, we included recruiter-level variables for variables that had attained high homophily earlier and were still included in our multivariate model. Recruiter-level variables that attained significance within the model would be retained, though ultimately none was significant, and all were omitted from the final model. Subsequently, we determined the need for fixed or random effects based on clustering determined in the first step. These adjustments would be retained if Akaike Information Criteria (AIC) tests indicated an improved model fit. None did, and thus all these effects were left out of the final model. Finally, we attempted an analysis including the effect of RDS individualized weights on model estimates; however, the small cell size of HIV-positive individuals caused the weights to inflate the standard errors and unstable estimates. Thus, the final multivariate model did not adjust for any clustering (due to non-significance) and was RDS unadjusted. This model was tested using generalized estimating equations, with HIV status as the dependent variable, using a binomial distribution and logit link function. We used a backwards stepwise approach with variables significant at *p*≤0.10 in the univariate analyses included. After ruling out multi-collinearity and interactions, the final model retained only variables that were statistically significant at 0.05 significance level. Degrees of freedom were set to varied across tests using the Satterthwaite approximation, which is recommended for data with a small sample size, an unbalanced covariance structure, or both.

### Power analysis

For the analysis of predictors of HIV, we computed statistical power using G*Power version 3.0.10. In computing statistical power, we assumed Fisher's exact test as the statistical test (since most of the predictor variables are categorical and have non-normal distributions), assumed an average small to medium effect size of 0.20 across the different predictors, and set the significance level to 0.05. Because one of the aims of this paper was to estimate the prevalence of HIV among MSM in Tijuana, we used actual data on prevalence to compute power. These assumptions yielded a value of 62% power to detect significant predictors of HIV.

## Results

### Recruitment dynamics

A total of 10 seeds (eight HIV-negative) recruited 216 participants. One seed did not recruit any participant. The longest recruitment chain was 13, and it recruited 59.2% of the sample. The median number of waves was five, and two-fifths of the sample was recruited at or after wave six. Most participants (78%) were recruited by friends, 14.7% by acquaintances, 4.7% by partners and 2.1% by family members. We excluded 15 candidates who failed to satisfy the following eligibility criteria: eight had not had sex with a man in the past 12 months; four were under the influence of alcohol or drugs at the time of enrolment; and three resided outside Tijuana. The resulting sample size was 201, and because seeds were not randomly recruited, we removed them from the analysis, yielding an *N* of 191 for all models.

### Demographics

Table 1 shows the unadjusted counts and the RDS-adjusted population proportions that we estimated using RDSAT 7.1 [25]. Mean RDS-adjusted age was 29.7 (range 18–65); 93.8% were estimated to be male and 5.8% were transgender. Sexual orientation was homosexual among an estimated three-fifths of the population (61.2%), with the rest identifying either as bisexual or heterosexual. Living with parents or non-partners was estimated as most common (40.4%), with more than half of the participants graduating from high school (50.1%), having a job (56.8%) and earning more than 3500 Mexican pesos ($280 US) per month (51.5%). We also estimated 27.5% ever to have travelled to the United States, but only 7.8% in the past year, with 3.6% having ever being deported.

**Table 1:** Characteristics of MSM in Tijuana, Mexico, 2012–2013 (*n*=191)

	Sample *n*	RDS population estimate	

		%	95% CI
Demographics			
Age, mean (SD)	30 (9.0)	–	–
Gender^a^			
Male	176	93.8	88.9–98.7
Transgender	14	5.8	1.0–10.6
Sexual orientation			
Homosexual	119	61.2	49.4–71.5
Bisexual/heterosexual	72	38.8	28.5–50.6
Graduated high school or more	104	50.1	39.4–61.3
Employed	112	56.8	47.0–68.9
Monthly income more than 3500 pesos (~280 dollars)	102	51.5	41.7–62.6
Travel to the United States			
Ever	55	27.5	19.0–38.7
Past year	22	7.8	3.7–13.0
Deported, ever	14	3.6	1.4–6.7
Jail, ever	54	30.2	19.8–40.5
Substance and alcohol use			
Ever used			
Marijuana	96	53.8	42.5–64.0
Cocaine	53	32.6	20.5–42.3
Methamphetamines	54	29.6	19.0–40.0
Amyl nitrate (poppers)	32	15.9	8.8–26.6
Used in the past month			
Marijuana	39	22.2	12.3–32.2
Methamphetamines	29	16.9	9.2–24.6
Amyl nitrate (poppers)	5	5.4	0.2–6.7
Cocaine	7	5.0	0.6–10.6
Used intravenous drugs, ever	10	6.3	1.2–12.8
Used drugs before sex, past 2 months	23	9.9	4.9–16.3
Hazardous alcohol use	77	33.3	24.2–43.7
Used alcohol before sex, past 2 months	76	34.7	25.3–43.9
Sex-related variables, past 2 months			
Sex partners, mean (SD)	6.1 (10.8)	–	–
Unprotected receptive anal, mean (SD)	2.6 (7.6)	–	–
Unprotected insertive anal, mean (SD)	3.9 (13.1)	–	–
Male sexual partners			
Spouse or live-in partner	51	31.1	20.5–44.4
Steady non-live-in partner	111	57.9	47.2–68.9
Casual partner	98	44.3	34.3–54.3
Anonymous partner	56	25.2	26.7–35.0
Female sexual partners	44	26.7	17.3–37.4
Sexual role in anal intercourse			
Insertive only	27	14.4	8.4–23.7
Receptive only	50	31.3	21.2–40.6
Both	88	41.9	31.4–52.6
Transactional sex			
Gave money for sex	44	16.7	9.0–24.7
Received money for sex	26	12.0	5.3–20.9
Had sex in a public venue	48	22.1	14.0–31.2
HIV and STI related			
HIV or STI counselling, past 2 months	28	12.0	6.0–18.5
Circumcised	33	14.8	8.8–22.6
Self-reported gonorrhoea, past 2 months	22	10.2	5.0–17.1
Tested for HIV, ever	135	63.7	53.2–74.5
Tested for HIV, past year	78	36.0	26.4–46.5
Prevalence			
HIV	33	20.2	12.5–29.1
Previously known HIV positive	2	0.5	0.0–1.7
Tested syphilis positive	14	6.1	2.2–12.2
Active syphilis (titer≥1:8)	4	2.5	0.2–6.8
Concurrent HIV/lifetime syphilis	6	4.1	0.6–9.7

a One participant refused to answer. RDS: respondent-driven sampling; SD: standard deviation; CI: confidence interval; STI: sexually transmitted disease.

### Substance and alcohol use

Marijuana (53.8%), cocaine (32.6%), methamphetamines (29.6%) and amyl nitrites (15.9%) were the drugs estimated to be the most commonly ever used among the MSM population in Tijuana. In the past month, estimated methamphetamine use was 16.9%. Only an estimated 6.3% ever injected drugs. A third (33.3%) were estimated to be hazardous alcohol users as determined through the AUDIT screening.

### Sexual activity

In the past two months, the mean number of male sex partners (after RDS adjustment for the overall population) was 6.1 (range 0–93), of unprotected receptive anal sex acts was 2.6, and of unprotected insertive anal sex acts was 3.9. We estimated that in the past two months, 31.1% had sex with a stable male sexual partner living in the same household, 57.9% with a stable male sex partner not living in the same household, 57.9% with a male casual partner, 25.2% with an anonymous male partner and 26.7% with a female partner. Also, in the past two months, almost a third (31.1%) were estimated to have had only receptive anal sex, 14.4% to have had exclusively insertive anal sex, and 41.9% to have had both. An estimated 16.7% paid for sex, with 12% receiving money in exchange for sex and a fifth (22.1%) having had sex in a public venue.

### HIV/STI prevalence and testing history

RDS analysis of the crude data resulted in an estimate that 63.7% of the population had ever been tested for HIV, with only 36% being tested in the past year, 12% participating in HIV or STI counselling in the previous two months and 14.8% being circumcised. The unadjusted HIV prevalence among study participants was 17.3% (*n*=33), with most (88.6%) being unaware of their HIV infection status. Of these newly diagnosed individuals, 25.8% reported that they had tested negative within the past year, suggesting newly acquired infections. Estimated HIV infection prevalence adjusted for RDS was 20.2% (95% CI: 12.5–29.1%). Prevalence of lifetime syphilis was estimated to be 6.1% and active syphilis (titer ≥1:8) to be 2.5%, with 4.1% testing positive for both lifetime syphilis and HIV. Gonorrhoea (10.2%) was the most common self-reported STI in the previous two months.

### Correlates of HIV infection

After bivariate analysis (Table 2), characteristics positively associated with HIV infection were the following: ever having used methamphetamines (OR=2.17), not having used alcohol hazardously in the past year (OR=0.34), not having used alcohol recently before or during sex (OR=0.23), not having had casual sex recently (OR=0.41), not having had sex in a public venue (OR=0.25) and testing positive for active syphilis (OR=4.17). Age, ever having used amyl nitrates, number of times having had unprotected receptive anal sex in the past two months and not having been tested for HIV in the past year were marginally (*p*<0.10) associated with HIV infection.

**Table 2:** Univariate analysis of correlates of HIV infection among MSM in Tijuana, Mexico (*n*=191)^a^

	HIV− (*n*=158)		HIV+ (*n*=33)		OR	*p*	95% CI
	
	*n*	%	*n*	%			
Demographics							
Age (per year increment)	29.0^b^	9.0^c^	32^b^	10^c^	1.03	0.10	0.99–1.07
Substance use							
Ever used methamphetamines	40	25.3	14	42.4	2.17	0.05	1.00–4.73
Ever used amyl nitrate	23	14.6	9	27.3	2.19	0.08	0.90–5.29
Hazardous alcohol use	70	44.3	7	21.2	0.34	0.02	0.14–0.83
Sex-related variables, past 2 months							
Alcohol before or during sex	71	44.9	5	15.2	0.23	0.01	0.08–0.67
Had casual sex	87	55.0	11	33.3	0.41	0.03	0.19–0.90
Had sex in a public venue	45	28.5	3	9.1	0.25	0.03	0.07–0.86
Unprotected receptive anal sex (per act increment)	2.0^b^	5.6^c^	5.2^b^	13.6^c^	1.04	0.05	1.00–1.09
HIV/STI related							
Tested for HIV in the past year	69	43.7	9	27.3	0.48	0.09	0.21–1.11
Tested syphilis positive	8	5.1	6	18.2	4.17	0.01	1.34–12.96

a Seeds (*n*=10) have been excluded from the analysis due to non-random sampling;

b mean;

c standard deviation. OR: odds ratio; CI: confidence interval.

In our multivariate model (Table 3), we found that relative to HIV-negative MSM, HIV-positive MSM were more likely to report ever having used meth and to have tested positive for active syphilis.

**Table 3:** Multivariate model of correlates of HIV infection among MSM in Tijuana, Mexico (*n*=191)

	AOR	*p*	95% CI	

			Lower	Upper
Ever used methamphetamines	4.33	0.01	1.42	13.19
Tested syphilis positive	2.24	0.05	1.02	4.92

CI: confidence interval. Seeds (*n*=10) have been excluded from the analysis due to non-random sampling. Model tested for clustering at shared recruiter, shared recruitment tree and shared colonia. None of the adjustments provided a better fit for the model; therefore, model is not adjusted for clustering. Also tested with recruiter-level variables for methamphetamine use, HIV status and syphilis status. All recruiter-level variables were not significant and thus not included in the model. AOR: adjusted odds ratio.

## Discussion

This study aimed to estimate the prevalence and correlates of HIV infection among MSM in Tijuana. Overall, we estimated a 20.2% HIV infection prevalence, which in the light of previous reports suggests an ongoing epidemic among this population [12, 29]. We also found that HIV-positive men were more likely to report lifetime meth use and more likely to have an active syphilis infection. This is consistent with previous research demonstrating the relationship between stimulant use and HIV in the United States [30, 31].

Among our findings, two stand out – HIV prevalence for this population is the highest among high-risk populations in Tijuana, and 89% of those infected were previously unaware of their status. While it may be that some of these men may have been unwilling to disclose their HIV-positive status, if our estimate is accurate, HIV prevalence among MSM in Tijuana is four times higher than among injection drug users [9], almost three times higher than among female sex workers [10] and four times higher than among male clients of female sex workers [11]. It is comparable, however, to the prevalence of HIV among MSM in neighbouring San Diego, California (18%), and slightly higher than the nationwide prevalence among MSM in Mexico (16.9%) [7].

Our data suggest that almost 9 out of 10 MSM in Tijuana who are HIV positive are unaware of their serostatus. We were unable to find a higher figure for an upper-middle-income country anywhere in the literature. In a recent study on unprotected anal sex among MSM in Tijuana, only 7% of participants reported being HIV positive, and 54.7% reported receiving an HIV test in the past year [24]. By contrast, our estimates show a higher HIV prevalence and lower frequency of HIV testing.

This study has several limitations. Due to its cross-sectional nature, we cannot assume causality of the factors correlated with HIV infection. As we used self-report to collect our data, recall bias could skew our results, although we tried to minimize this by limiting the recall period for most questions to two months, and we do not believe there was differential reporting among our subgroups. As we surveyed on sensitive topics, social desirability bias may also have led to underestimates of certain high-risk behaviours. However, we aimed to minimize this by using well-trained interviewers trusted by the local community. Data collection may have also been affected by high participant burden caused by the long survey and interview. Finally, even though we used RDS, which theoretically increases the generalizability of our estimates to the population, controversies exist concerning its precision [32], and consensus is lacking on how best to handle multivariate analysis. We followed a process that attempts to correct for possible distorting effects of recruitment patterns (homophily and clustering), although none of those effects proved significant in our models. The relatively small sample and small number of HIV-positive individuals caused lower power than desired (0.62 vs. 0.80); therefore, the results regarding predictors of HIV should be interpreted with caution. Given all these considerations, our findings may not be representative of the MSM population in Tijuana or elsewhere in Mexico. However, they do indicate the urgent need for further intervention research with this population.

A major increase in the HIV testing rate is critical to curbing the epidemic and improving health and life outcomes among this population. With their status unknown, MSM in Tijuana are not being sufficiently linked to care. The high prevalence of HIV in this population, the low rate of awareness of infection status and the risk behaviours engaged in could be markers for a high community viral load. This situation will persist until HIV+MSM in Tijuana are identified, linked to care and placed on antiretroviral medication. In addition, intervention research is needed to reduce risky behaviour and increase safer sex among MSM. Peer-led interventions to reduce unprotected anal sex among MSM appear to be particularly efficacious, though more research is needed using randomized controlled designs [33].

Behaviour-change interventions to prevent HIV among MSM in Tijuana should also focus on substance use. MSM who become infected with HIV and continue to use meth are more likely to miss doses of antiretroviral medication or to stop taking them entirely, less likely to have access to medical care and more likely to report high-risk sexual behaviours [13]. Behavioural interventions that use cognitive-behavioural therapy [34, 35], contingency management [36] and text messaging [37] appear promising in some trials to reduce methamphetamine use and HIV-risk-related behaviours among MSM but higher degrees of efficacy and consistency are desirable.

## Conclusions

There is a growing HIV epidemic in Tijuana, Mexico, a city on the US–Mexico border that may affect epidemics on both sides of the border. Compared to research with other high-risk groups in Tijuana such as female sex workers and injection drug users, and compared to research on MSM in other parts of the world, very little research has been done to examine HIV in Tijuana. We conducted a study using RDS to estimate prevalence and correlates of HIV among MSM in Tijuana. Our data suggest that one in five MSM in Tijuana are infected with HIV, and that the majority of these men are unaware of their HIV status. There is a need to increase HIV testing uptake among MSM in Tijuana, and to address substance use and co-occurring STIs to help prevent HIV in this high-risk and vulnerable population.
